# Transcriptome analyses of primitively eusocial wasps reveal novel insights into the evolution of sociality and the origin of alternative phenotypes

**DOI:** 10.1186/gb-2013-14-2-r20

**Published:** 2013-02-26

**Authors:** Pedro G Ferreira, Solenn Patalano, Ritika Chauhan, Richard Ffrench-Constant, Toni Gabaldón, Roderic Guigó, Seirian Sumner

**Affiliations:** 1 Center for Genomic Regulation, Universitat Pompeu Fabra (CRG-UPF), Doctor Aiguader, 88, 08003 Barcelona, Catalonia, Spain; 2 Institute of Zoology, Zoological Society of London, Regent's Park, NW1 4RY, UK; 3 The Babraham Institute, Babraham Research Campus, Cambridge, CB22 3AT, UK; 4 Centre for Ecology and Conservation, Biosciences, University of Exeter, Tremough, Penryn, TR10 9EZ, UK; 5 Current address: School of Biological Sciences, University of Bristol, Woodland Road, Bristol, BS8 1UG, UK; 6 Current address: Department of Genetic Medicine and Development, University of Geneva Medical School, 25 Rue Michel Servet 1, 1211 Geneva, Switzerland

## Abstract

**Background:**

Understanding how alternative phenotypes arise from the same genome is a major challenge in modern biology. Eusociality in insects requires the evolution of two alternative phenotypes - workers, who sacrifice personal reproduction, and queens, who realize that reproduction. Extensive work on honeybees and ants has revealed the molecular basis of derived queen and worker phenotypes in highly eusocial lineages, but we lack equivalent deep-level analyses of wasps and of primitively eusocial species, the latter of which can reveal how phenotypic decoupling first occurs in the early stages of eusocial evolution.

**Results:**

We sequenced 20 Gbp of transcriptomes derived from brains of different behavioral castes of the primitively eusocial tropical paper wasp *Polistes canadensis*. Surprisingly, 75% of the 2,442 genes differentially expressed between phenotypes were novel, having no significant homology with described sequences. Moreover, 90% of these novel genes were significantly upregulated in workers relative to queens. Differential expression of novel genes in the early stages of sociality may be important in facilitating the evolution of worker behavioral complexity in eusocial evolution. We also found surprisingly low correlation in the identity and direction of expression of differentially expressed genes across similar phenotypes in different social lineages, supporting the idea that social evolution in different lineages requires substantial *de novo *rewiring of molecular pathways.

**Conclusions:**

These genomic resources for aculeate wasps and first transcriptome-wide insights into the origin of castes bring us closer to a more general understanding of eusocial evolution and how phenotypic diversity arises from the same genome.

## Background

Phenotypic plasticity is a fundamental biological process that allows organisms to adapt to changes in their environment [1,2]. Examples of plastic phenotypes include insect castes [3,4], horn-polyphenic beetles [5] and sex differences [6], where they play a crucial role in shaping the ecology and evolution of species and ecosystems. Understanding how alternative phenotypes arise from the same genome is one of the most challenging questions in modern biology [1,2].

The most impressive examples of phenotypic plasticity are found in the eusocial Hymenoptera (ants, some bees and some aculeate wasps), where specialized reproductive or non-reproductive phenotypes (castes) play a pivotal role in the ecological success of these insects [7]. Eusocial insect castes usually arise through differential expression of shared genes [8,9], but studies to date have been largely restricted to the highly eusocial bees and ants, where caste phenotypes are evolutionarily derived, being greatly modified from their ancestral state (for example, with morphological adaptations that are established early in development). Thus, some of the molecular processes underlying the origins of castes are likely to differ substantially from those underlying highly eusocial species [10,11]. Understanding how castes first arise is best studied in primitively eusocial species, but little is known about their molecular evolution, particularly in the aculeate wasps [12-15]. Thus, there is a need for comprehensive gene-level studies on primitively eusocial wasps.

In the evolution of eusociality, behavioral and physiological traits of the non-social ancestor become decoupled into complementary queen and worker phenotypes [16], but we understand little about the molecular patterns and processes by which this originates and to what extent molecular ground plans are re-organized. These questions are of general biological importance in understanding the evolution of phenotypic diversity [1,2]. Empirical assessment of how gene transcription is decoupled into alternative phenotypes has been difficult to obtain because it requires unbiased quantification of gene expression (of both known and novel (undescribed) genes) across whole genomes [17]. Microarray analyses of the highly eusocial honeybee (*Apis mellifera*) and fire ant (*Solenopsis invicta*) suggest marked functional decoupling of molecular processes in queens and workers, involving large numbers of genes [18-21]. Little is known about the patterns and processes of transcriptional decoupling for castes in primitively eusocial insects [12,14]. A simple prediction is that patterns of transcriptional and functional decoupling should be less pronounced than in highly eusocial species, because phenotypes are in the first stages of merely behavioral decoupling [9,12].

Processes of phenotypic decoupling at the molecular level may occur via several mechanisms [5,22]. Conserved toolkits of ancestral molecular processes may be redeployed to regulate similar alternative phenotypes across species and lineages [23-26]. In eusocial insects, conserved toolkit genes appear to be differentially expressed in castes across species, including bees and wasps [12,27]. An alternative, untested prediction is that at least some of the molecular processes underlying social phenotypes, in different social lineages and among different levels of social complexity, will differ substantially. This is because extensive molecular and developmental re-wiring or new gene evolution may be required for the loss of phenotypic plasticity and elaboration of phenotype-specific traits [10,28]. There may also be rapid evolution of genes associated with the emergence of alternative phenotypes [6,17,29], and there is evidence for this in the highly eusocial honeybees and fire ants [30,31]. Finally, the importance of novel genes (that is, previously undescribed genes that lack any detectable protein-coding homologues from existing sequence data [32]) in the evolution of phenotypic innovations has recently emerged in animals as varied as yeast [33], hydra, reptiles [34-36], and also honeybees [37]. To date the eusocial insect literature has largely focused on conserved molecular pathways for social behavior [12,26,27,38,39], and the relative roles of the different mechanisms of phenotypic decoupling are unknown.

**Figure 1:**
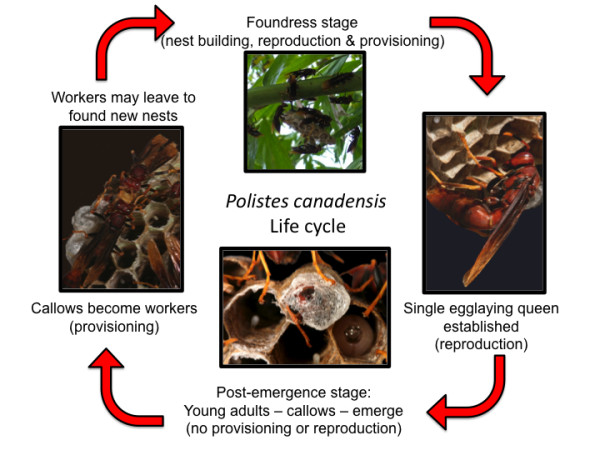
**Phenotypes produced through the life cycle of the tropical paper wasp *Polistes canadensis***. Queens and workers show specific behaviors - reproduction or provisioning, respectively. Foundresses show both worker and queen behaviors, and additionally build new nests, whilst callows (newly emerged females; <2 days old) exhibit none of these behaviors. Colonies are founded, and males and females produced, throughout the year with no seasonal diapause.

We sequenced over 20 Gbp of genome-wide caste-specific transcripts from the primitively eusocial, tropical paper wasp *Polistes canadensis *using high-throughput sequencing technologies. Castes in *Polistes *differ only in behavior and are determined through differential gene expression during adulthood [12-14], making *Polistes *important models for studying the early stage in the evolution of eusociality and phenotypic plasticity [40]. *Polistes *originated in the tropics, and the secondary adaptations to diapause found in temperate species (for example, [41]) will be absent in *P. canadensis *[42]. Typically a few foundresses build and provision new colonies (Figure 1). One foundress becomes the primary egg-layer (queen). Emerging adults help raise the offspring of their mother queen, but retain the ability to become egg-layers [43-45]. Thus, *Polistes *castes represent an early stage of phenotypic decoupling in the evolution of eusociality [16].

Here, we generate a genome-wide catalogue of aculeate wasp genes expressed in adult females to provide a resource for genomic analyses. We then conduct the first RNA-seq analyses of caste-biased expression in a wasp and primitively eusocial insect to test the above hypotheses on the patterns and processes of molecular decoupling of alternative phenotypes at the early stages in the evolution of eusocial behavior. These datasets allow a first simultaneous assessment of the role of conserved genetic toolkits, novel genes and gene evolution when social behavior first evolves. We also use our data to re-assess the phylogenetic relationships between the three aculeate Hymenoptera subfamilies (bees, wasps and ants), which is vital information for interpreting common processes and lineage-specific novelties underlying the evolution of alternative phenotypes. Recently, there has been much debate over the relationships of aculeate bees, wasps and ants, challenging the morphology-based view that Vespoidea (ants plus aculeate wasps) are monophyletic [46,47]. Genome sequences (or similar catalogue of genes) for aculeate wasps will help resolve this controversy and provide a basis for interpreting comparative data on social evolution.

## Results and discussion

### Gene assembly for an aculeate wasp

We first generated a reference assembly of the genes expressed by *P. canadensis *adult female phenotypes (queens, workers, foundresses and callows) (Figure 1; Section 1 in Additional file 1) by sequencing normalized 454 libraries from phenotype-specific pools (37 individuals in total; Section 2 in Additional file 1). Using transcriptome rather than genome sequencing data to provide a gene set is a powerful and accessible approach for initiating genomic analyses on non-model organisms. Library normalization meant that we were able to capture transcripts at all levels of the expression spectrum. The longer read lengths provided by 454 technology and the high coverage of these datasets increase the likelihood of full transcript assembly. The 454 reads were assembled into a reference transcriptome set of 26,284 isogroups - henceforth referred to as 'genes' for simplicity (Section 2 in Additional file 1; Additional file 2). This gene set included 98% of the core eukaryotic genes with equal coverage for each phenotype (Section 3 in Additional file 1). Additionally, sequences were 100% identical to existing overlapping Sanger sequences for this species (n = 36 genes) [14]. Genes were equally spread across honeybee chromosomes and included 94% of publicly available ESTs for the temperate paper wasp *Polistes metricus *(n = 422 genes [12]; Section 3 in Additional file 1). Although this assembly is restricted to the genes expressed in adult females, it provides an important step forward in generating a comprehensive genome-wide sequence resource for aculeate wasps.

We characterized in detail the sequence and expression features for two groups of genes. The first group corresponds to the set of genes with detectable homology to protein sequences of any species in the comprehensive GenBank non-redundant (NR) database. We identified homologs for 37.4% of the total *P. canadensis *genes (n = 9,839), which were then annotated using Gene Ontology (GO; Table 1; Section 4 in Additional file 1; Additional files 3 and 4): 71% of best hits were with the honeybee, which reflects the wealth of genomic resources for this species rather than any close phylogenetic relationship (see below). This level of homology is comparable with data for the temperate wasp *P. metricus *where 39% of the approximately 391K sequence fragments had putative orthologs [13]. In comparison, 60% to 70% of genes in the genomes of other social insects (ants and bees) had homology with sequences in other insects [48,49].

**Table 1:** Number of best BLAST hits between the genes of sequenced genomes for aculeate hymenopterans

Species	*P. canadensis*	*A. mellifera*	*S. invicta*	*C. floridanus*	*H. saltator*	*N. vitri-pennis*
*P. canadensis*	22,460 (100%)	7,040 (31.3%)	5,548 (24.7%)	6,501 (28.9%)	5,880 (26.2%)	4,416 (19.7%)
*A. mellifera*	5,764 (52.1%)	11,062 (100%)	7,093 (71.4%)	8,197 (74.1%)	6,769 (61.2%)	5,366 (48.5%)
*S. invicta*	4,662 (28.2%)	6,293 (38.0%)	16,522 (100%)	8,864 (53.6%)	8,341 (50.5%)	4,935 (29.9%)
*C. floridanus*	5,422 (31.8%)	7,129 (68.6%)	11,741 (68.6%)	17,064 (100%)	12,492 (73.2%)	5,552 (32.5%)
*H. saltator*	4,913 (26.5%)	6,798 (36.6%)	10,591 (57.0%)	9,247 (49.8%)	18,564 (100%)	5,667 (30.5%)
*N. vitripennis*	3,861 (20.5%)	5,377 (28.6%)	6,144 (32.6%)	6,600 (35.0%)	5,725 (30.4%)	18,822 (100%)

Absolute number (and percentage of genes) shared between each pair of species are given. Annotations used for each species are: Official Gene Set (OGS)1.2 for the parasitoid wasp *Nasonia vitripennis*, OGS3.3 for the ant *Harpegnathos saltator*, OGS3.3 for the ant *Camponotus floridanus*, OGS2.2.3 for the ant *Solenopsis invicta*, pre-release of OGS2.0 for the honeybee *Apis mellifera*, and the longest ORFs (putative coding genes only - that is, excluding 3,824 potential long non-coding RNAs) for *Polistes canadensis *from our RNAseq datasets. Rows represent the source species, which are compared with the test species in the columns. For instance, of the 11,062 genes found in *A. mellifera*, 5,764 have significant hits with genes found in *P. canadensis*, corresponding to 52.1% of the total 11,062 genes. *P. canadensis *shares 25 to 31% of genes with the other aculeate hymenopterans, but only 19.7% with the parasitoid *N. vitripennis*. Notably, *P. canadensis *does not share more genes with the ants than the bee, in agreement with our maximum-likelihood phylogenetic analysis (Figure 3a).

**Figure 2:**
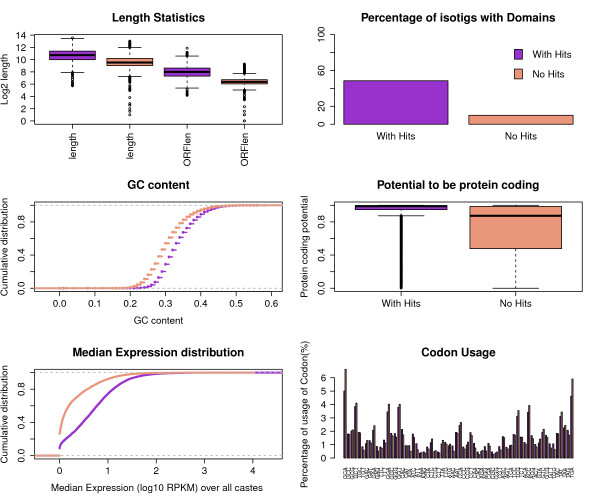
**Sequence and expression characterization of the transcripts with and without detected homologs**. (a) Length and longest ORF length statistics. **(b) **Percentage of transcripts with known protein domains. **(c) **Distribution of GC-content. **(d) **Potential to be protein coding. **(e) **Distribution of the median expression across all the castes. **(f) **Codon-usage frequencies. RPKM, reads per kilobase per million.

The second group of genes (n = 16,445; 62%) lacked detectable homology with protein sequences in NR databases, and will be referred to here as putative novel genes as they differ significantly in sequence from any described sequences to date (Section 5 in Additional file 1). These include taxonomically restricted genes, and they may be derived from ancestral coding genes, or arise *de novo *from noncoding ancestors [32]. Overall, transcripts with homology were longer (with-hits group, 1,718 ± 1,343; no-hits group, 736 ± 768) and had longer ORFs (with-hits group, 256 ± 205; no-hits group, 83 ± 36) than those without (Figure 2a). By scanning the sequences for known protein domains with HMMscan [50] we detected domain homology in 48% of the sequences with NR hits and only 9.9% in the group without hits (Figure 2b). GC content was also lower in the no-hits group (mean GC with-hits, 0.337: no-hits, 0.306; Figure 2c). Analysis of protein coding potential with PORTRAIT [51] revealed a significantly lower potential for protein coding in the group of isotigs without hits (Figure 2d). We then examined whether the novel isotigs could potentially be long non-coding RNAs (lncRNAs). First, we looked more closely at the distribution of isotigs that are longer than 300 bp and have an ORF shorter than 90 amino acids (96% of the *A. mellifera *transcripts have an ORF greater than 90 amino acids). The vast majority of these isotigs lacked homology (n = 12,751 with no homology; n = 1,162 with homology), suggesting that the putative novel isotigs had shorter ORFs. Next, we imposed a PORTRAIT score lower than 0.5 (at which a sequence is more likely to be non-coding than coding), which yielded more isotigs in the set lacking homology (n = 4,096), than those with (n = 314). Finally, we imposed the condition of no homology with known protein domains, which again yielded more isotigs in the set without homology (n = 3,824) than with (n = 262). These latter two results suggest lower protein coding potential in the putative novel isotigs. The distribution of the median expression across all castes shows that, in general, isotigs with hits have higher expression values than those without (Figure 2e; but see also expression analysis below). The pattern of codon usage was similar in both groups (*P *= 1, two sample *t*-test; Figure 2f).

The characteristics of isotigs lacking homology - namely shorter ORFs, lower protein coding potential, lower GC content and lower expression values [52] - suggest that a large proportion of novel isotigs correspond to non-protein coding transcripts. But low sequence conservation across species, lack of genomic information for aculeate wasps and the typical tissue specificity of the non-coding RNAs [52] may also explain the novelty of these genes.

We also verified that the abundance of novel genes was not an artifact resulting from assembly of transcript fragments rather than complete transcript sequences (Section 6 in Additional file 1). Our analyses suggested that less than 10% of genes were affected by this, and thus do not alter significantly our main analyses (below). However, without a genome sequence we cannot exclude the possibility that some genes may represent incomplete assemblies.

### Paraphyly of Vespoidea

**Figure 3:**
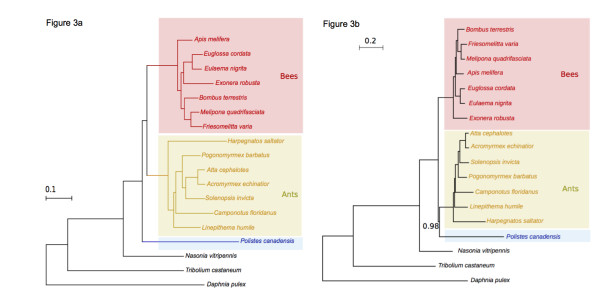
**Phylogenetic relationships across hymenopterans with sequenced genomes or transcriptomes**. **(a) **Phylogeny inferred from a maximum-likelihood analysis of a set of 93 conserved proteins (see Materials and methods). The coleopteran *Tribolium castaneum *and the crustacean *Daphnia pulex *are used as out-groups. *P. canadensis *(blue) appears basal to a clade formed by ants (yellow) and bees (red), suggesting Vespoidea (ants + aculeate wasps) are not monophyletic. Non-maximal support values are indicated in the corresponding branches, and are based on Shimodaira-Hasegawa-like approximate likelihood ratio tests. This topology is statistically more supported than the alternative scenario in which *Polistes *is the sister group of ants (see Materials and methods). **(b) **Consensus tree from the Bayesian analysis places *Polistes *as sister group to the ants, supporting the classical scenario. All nodes received maximal posterior probability except the one supporting the monophyly of Vespoidea. Other differences between the maximum-likelihood and Bayesian analyses concern the internal branching order within bees and ants.

Long-standing analyses based on the fossil record, cladistics and phylogenetic analyses of the aculeate Hymenoptera place ants and aculeate wasps as a monophyletic clade - the Vespoidea - with the bees as a sister group. This has received mixed support from recent molecular analyses [47,53-55], but these studies were limited to a maximum of four genes and sparse data matrices. High-throughput transcriptomics data are a reliable source of phylogenetic information [56]. Our transcriptome affords a first assessment of the monophyly of Vespoidea using a large and complete data matrix. We analyzed 93 conserved one-to-one orthologs of *P. canadensis *genes, derived from 196 single-copy insect phylogeny markers in the eight fully sequenced hymenopteran genomes [57] and nine bee transcriptomes [38] (Additional file 5). A maximum-likelihood (ML) analysis of these genes across all 32 insect species placed *Polistes *as the basal, sister group to the bees and ants (Figure 3a). This topology was significantly more supported (*P *< 0.05, Shimodaira-Hasegawa test) than the earlier proposal that Vespoidea (ants and *Polistes *in our study) are monophyletic. This result is consistent with the levels of gene sharing across aculeates (Table 1), where *P. canadensis *does not share more genes with ants than bees, as would be expected if wasps and ants were monophyletic.

We further tested the phylogenetic relationships using a Bayesian framework. In contrast, this gave greater support to the classical view of aculeate wasps and ants being monophyletic (Figure 3b; Section 10 in Additional file 1). The reason for the discrepancy in the results of these two methods is likely to be due to the wasp clade being the most poorly represented in our analysis (with only one species), and moreover, it is the clade with the longest branch. Our dataset offers a first chance to examine subfamily relationships across large numbers of genes but, clearly, phylogenomic data on more species of aculeate wasps are required to determine whether the term 'Vespoidea' should be dropped, or reclassified as paraphyletic. This finding would have important general implications for our understanding of eusociality as it would suggest that bees and ants shared an aculeate wasp-like common ancestor, that ants are wingless wasps [58], and that bees are wasps that lost predacious behaviors. Primitively eusocial wasps may therefore hold some important clues to the early stages of eusociality, if they are the basal descendants of non-aculeate Hymenoptera.

### Transcriptional and functional decoupling at the origin of alternative phenotypes

We next conducted RNA-seq analyses of phenotype-biased expression to provide a first genome-wide look at molecular decoupling of alternative phenotypes at the early stages of eusocial evolution. We quantified genome-wide transcription in individuals (n = 2 to 12 per phenotype) from each of the four adult female phenotypes by sequencing at least 3 Gbp of Illumina short-reads of brain cDNA per phenotype (Additional files 6, 7, 8 and 9). These samples appeared to capture most transcription across the genome since 99.8% of the 26,284 genes in the reference transcriptome were detected in our pooled Illumina dataset (Section 7 in Additional file 1).

We identified genes that were differentially expressed between phenotypes using NOIseq [59]. Of the 26,284 genes, 2,442 (9.3%) were differentially expressed in any one phenotype (Table 2; Section 8 in Additional file 1; Additional file 10). This suggests that a very small part of the adult female transcriptome is decoupled to produce alternative phenotypes in this species. This is similar to that detected in the temperate wasp *P. metricus*, where 12% (n = 389) of genes on a microarray were caste-biased [12]. Because RNA-seq is a new methodology, we also confirmed that the number of differentially expressed genes was not artificially elevated due to gene fragmentation (Section 9 in Additional file 1). In a microarray study on whole body samples of the highly eusocial fire ant *Solenopsis invicta*, the proportion of examined genes that were differentially expressed between adult queens and workers (19% of 14,467 genes) [21] was twice that of *Polistes*. RNA-seq analyses on highly eusocial species like the fire ant are required to explore these patterns of transcriptional decoupling further.

**Table 2:** Isotig and **i**sogroup differential expression statistics for the four adult female phenotypes in *P. canadensis*

Phenotype	Up-regulated (q > 0.6) isogroups (isotigs)	Isogroups with recognized homologs	Isogroups with annotated homologs	Down-regulated (q > 0.6) isogroups (isotigs)	Isogroups with recognized homologs	Isogroups with annotated homologs
Queen	47 (67)	39 (82.9%)	20 (42.5%)	402 (522)	63	38
Worker	2,222 (2,924)	412 (18.5%)	238 (10.7%)	84 (109)	65	38
Foundress	11 (15)	8 (72.7%)	4 (36.4%)	2,389 (3,340)	776	507
Callow	162 (238)	142 (87.6%)	112 (69.1%)	1,454 (1,779)	322	194
Total	2,442 (3,244)	596 (24.4%)	370 (15.2%)	4,329 (5,750)	1,311	858

We show the number of isogroups and isotigs found as differentially expressed by NOISeq at a threshold q > 0.6; number and percentage of differentially expressed genes with significant homologs recognized in GenBank non-redundant databases; the number and percentage of annotated homologs; and values across all castes are given in the final row of the table. Distribution of up-regulated isogroups, recognized homologs and annotated homologs are highly significantly different between queens and workers (chi-square test; respective chi-square values: 1,566 (1d.f.), *P *< 2.2e-16; 4,795 (1d.f.), *P *= 9.1 × 10^-13^; 5,382 (1d.f.), *P *< 2.2e-16).

**Figure 4:**
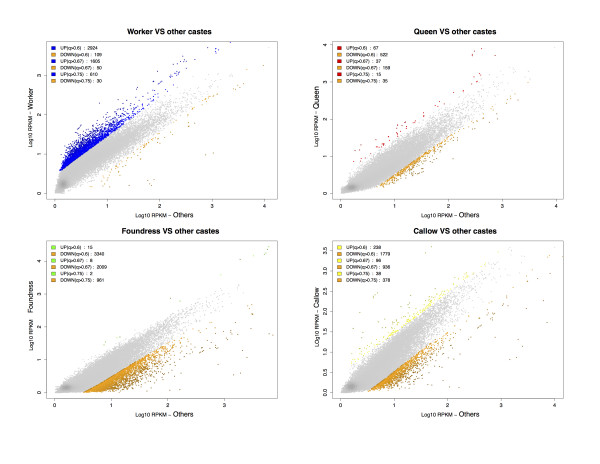
**Differentially expressed genes in the four phenotypes**. Distribution of gene expression (log10 transformed RPKMs (reads per kilobase per million)) between one phenotype versus the others, with differentially expressed transcripts highlighted. Colours represent different probability values. Darker regions represent higher transcript density. Number of differently expressed transcripts at different NOISeq [59] probability values are also presented.

**Figure 5:**
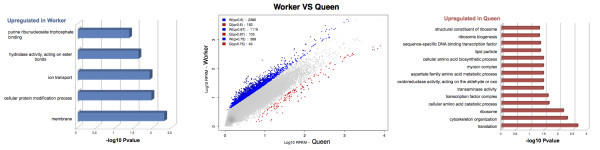
**Differentially expressed genes in queen versus worker comparison and GO terms over-represented in each caste**. Only the most specific terms, as obtained from the Blast2Go analysis, are represented. See Additional file 5 for the expanded set.

Workers appear to upregulate significantly more caste-biased genes (n = 2,224; 91% of caste-biased genes) than other phenotypes (callows, foundresses and queens) (Figure 4 and Table 2). This result holds for all different NOIseq [59] selection thresholds (Figure 4) and is independent of the number of biological replicates per phenotype analyzed (Section 8 and Table S2 in Additional file 1). Our original aim was to examine decoupling in queens and workers. We therefore re-analyzed the data using only these two phenotypes and found an even greater asymmetry with workers up-regulating 93.1% (n = 1,797) of 1,909 worker/queen caste-biased genes (queens up-regulate only 112 genes; Table 3 and Figure 5). Upregulation of caste-biased genes in workers, rather than queens, at the early stages of caste evolution may facilitate the expansion of worker trait complexity, typified by workers in the highly eusocial descendants of primitively eusocial species. These results support the emerging picture that much of phenotypic evolution occurs in workers, rather than queens [21,37], and provide the first suggestion that molecular evolution of the worker caste is in place early in social evolution.

**Table 3:** Isotig and isogroup differential expression in the comparison of only queen and worker castes

Caste	Up-regulated (q > 0.6) isogroups (isotigs)	Isogroups with recognized homologs	Isogroups with annotated homologs	Putative lncRNAs (total identified = 3,824)
Queen	112 (163)	91 (55.8%)	51 (45.5%)	6 (5.3%)
Worker	1,797 (2,380)	347 (19.3%)	217 (12.1%)	339 (18.86%)

Significant differences between queens and workers in the numbers of isogroups and isotigs found as differentially expressed by NOISeq at a threshold of q > 0.6 (chi-square value, 1,083 (1d.f.); *P *< 2.2e-16); number and percentage of differentially expressed genes with significant homologs recognized in GenBank NR database (chi-square value 102 (1d.f.); *P *< 2.2e-16); the number and percentage of annotated homologs (chi-square value, 70 (1d.f.); *P *< 2.2e-16); number and percentage of putative lncRNAs (chi-square value, 236 (1d.f.); *P *< 2.2e-16) are given.

Interestingly, foundresses and callows accounted for the majority (89%) of down-regulated genes. Some of these could be attributed directly to a corresponding up-regulation in workers: specifically, 55% and 75%, respectively, of the down-regulated genes in foundresses and callows. But, the remaining foundress and callow down-regulated genes were not upregulated in other castes. This is also reflected in the GO classification (Additional file 5), where none of the 22 GO terms over-represented among the down-regulated genes in foundresses and only 5 of the 23 terms over-represented among the down-regulated genes in callows were up-regulated in workers. Although preliminary, these results raise some important questions: they suggest that callows are born neither worker- nor queen-like, but perhaps instead as an undifferentiated state, as expected in a species where all females retain full reproductive totipotency; down-regulation in callows may also reflect maturation processes that take place in the first days after emergence as an adult. Foundresses are thought to be hopeful reproductives, co-founding a nest in the hope of becoming the dominant egg-layer (queen); our data suggest that foundresses are quite different from established queens, even though they fit the queen phenotype in all other respects (that is, they are active egglayers, rarely absent from the nest, and are behaviorally dominant). Taken together, these results highlight how the definition of a phenotype in this species is more complex than a simple distinction between queens and workers.

Using the 268 genes differentially expressed between queens and workers that could be annotated (Table 3), we then looked for any signs of functional specialization between these castes. Of the GO terms corresponding to these genes (Additional file 4), 2.4% (53 out of 2,242) were significantly enriched between queens and workers (Additional file 5; 40 terms (1.7%) over-represented in queens, 13 terms (0.57%) over-represented in workers). Although many of our genes did not have any functional information, there are some interesting first insights from these analyses. The putative lack of substantial functional specialization between castes in *Polistes *contrasts with the marked functional specialization in highly eusocial species, such as honeybees [19]. This may reflect selection on females to retain reproductive totipotency in primitive societies, like *P. canadensis*, in order that they can exploit alternative reproductive strategies [43], and minimize the risks of specialization associated with small colony size [60]. General terms overrepresented in workers include cellular protein modification processes, membrane and ion transport, which may suggest elevated cellular activity in worker brains. Among the over-represented terms in queens, several candidates associated with cell structure and cytoskeleton were found, such as homologs of myosin chains, actin isoforms and troponin, which may be associated with synaptic plasticity and memory [61]. However, because so few of the genes up-regulated in workers could be annotated, and because of the lack of significant levels of enrichment between castes, these inferred functional differences between castes should be taken with caution and require further analyses once some functionality can be assigned to the unknown genes.

### Role of novel genes in the evolution of alternative phenotypes

We explored the hypothesis that novel genes (those lacking homologs in NR databases; see above) may play an important role in the early stages of phenotypic decoupling in *P. canadensis*. The importance of novel genes was recently highlighted in organisms as varied as *Hydra *to reptiles for their contribution to phenotypic diversity, through generating both behavioral and morphological variation [34-36]. Of the 2,442 up-regulated caste-biased genes (Table 2), 24.4% (n = 596 genes) had known homologues, indicating that both novel and described genes are important in *P. canadensis *social behavior. Interestingly, 81.5% of worker-biased genes lacked homologs, compared to only 17.1% of queen-biased genes (Table 2). Foundresses and callows show similar levels of homology to queens. Lack of homology cannot be attributed to fragmentation or other anomalies arising from using transcriptome data (Section 6 in Additional file 1) and they are also valid for the stricter comparison of queens versus workers (Table 3). These results are exactly in line with Johnson and Tsutsui [37] and Barchuk *et al*. [62], who found an over-representation of novel (taxonomically restricted) genes up-regulated in honeybee workers (adults [37] and brood [62]), and suggested that these novel genes may facilitate expansion of worker behavioral complexity. Our data provide the first suggestion that novel gene transcription may be important at the early stages of worker evolution, confirming the potentially broad role of novel genes in eusocial evolution [37] and adding to the emerging general importance of novel genes in the evolution of phenotypic diversity [34-36]. It is important to note that microarray methods (as used in the honeybee studies) are largely biased towards conserved genes [37], as sequence conservation facilitates gene prediction. Thus, RNA-seq methods as used in our study offer new potential to explore the role of putative novel genes in phenotypic evolution.

Our ability to interpret what these novel genes are is limited without genome and proteome analyses, which is beyond the scope of this study. But, we explored the hypothesis that many of the caste-biased novel genes were in fact non-coding RNAs, as identified from our whole transcriptome analysis (above). Interestingly, we found a significant difference in the proportion of novel transcripts that were putatively lncRNAs, with an over-representation in workers relative to queens (Table 3). lncRNAs are thought to play a role in gene regulation [63], but also may be evidence of *de novo *gene origin [33]. Future work will determine the significance of these processes in worker, rather than queen, evolution.

### Conserved molecular toolkits associated with alternative phenotypes

Social lineages evolve from non-social ancestors [64]. Alternative phenotypes (social insect castes) may therefore evolve through the decoupling of conserved sets of genes that regulated changes in hormone titers underlying the provisioning and reproductive phases of the non-social ancestor [16]. A prevailing hypothesis is that changes in the patterns of expression of these ancestral ground-plan genes could account for caste evolution across social taxa [26,28]. Using a candidate gene approach, sets of such 'toolkit' genes and molecular processes have been shown to have conserved roles in caste regulation across bees and wasps [12,27,38]. We also found some evidence of this in *P. canadensis*: vitellogenin (four isogroups), insulin (one isogroup) and major royal jelly protein (two isogroups) were queen-biased; juvenile hormone (two isogroups) and methyltransferases (two isogroups) were worker-biased. However, other toolkit genes (hexamerin, *Malvolio, Amfor*, P450) were present in our dataset, but were not identified as significantly caste-biased (Additional file 4) [12,27,65,66].

As a genome-wide, unbiased representation of genes expressed by alternative phenotypes in wasps, our datasets also offer the opportunity to test to what extent a conserved molecular toolkit in general explains alternative social phenotypes across eusocial taxa [12,27]. Interestingly, we found little correlation in the general identity and direction of expression of genes underlying castes in other eusocial insects, as identified from microarray analyses. For example, only 6.5% of honeybee (*A. mellifera*) caste-biased genes [19] were caste-biased in *P. canadensis *and there was no consistency in the direction of expression. Of 50 cDNAs predictive of honeybee worker behavior [18], only 8 were found in *P. canadensis *and they were not significantly worker biased (Additional file 5). There was no correlation in direction and identity of gene expression in castes of the highly eusocial fire ant *S. invicta *(r ~ 0) [21]. Finally, only 22 of the 423 caste-biased genes of the temperate wasp *P. metricus *[12] were also caste-biased in *P. canadensis*, and they showed a low correlation in the direction of phenotype-specific expression between species (Pearson correlation of 0.25 for worker, 0.14 for queen, 0.24 for foundress and 0.23 between gyne and callow; Additional file 5). This was unexpected since these two species are close relatives, share a recent common ancestor, and have similar social and behavioral traits. Differences in ecology of tropical and temperate *Polistes*, and possible secondary adaptation to temperate living [42], may contribute to these differences. These analyses suggest that the social toolkit represents a small portion of the genome involved in phenotype decoupling of *P. canadensis*, and that there is much lineage and ecology-specific transcription underlying caste differences. This supports the hypothesis that the genes underlying castes in different social lineages will not necessarily be conserved, because extensive molecular and developmental re-wiring may be required in the evolution of caste commitment and eusociality [10,11].

### Insights into gene evolution in alternative phenotypes

Rapid evolution of genes has been associated with alternative phenotypes in a range of animals [6,17,29]. Caste-biased genes evolve rapidly in the honeybee and fire ant [30,31], possibly because fast-evolving genes are preferentially recruited into caste-biased gene expression [30]. Interestingly, we found no evidence of this in *P. canadensis *(Section 10 in Additional file 1; Additional file 5), suggesting that at the early stages of phenotypic decoupling genes are maintained through purifying selection. This agrees with social evolutionary theory that predicts that females retain the ability to exploit alternative reproductive opportunities (for example, to switch from worker to queen) to maximize individual-level inclusive fitness at the early stages of sociality [43]. Selection may be relaxed after workers commit to a non-reproductive pathway, as found in highly eusocial species [10,28], resulting in gene evolution, new genes and new gene networks [31,67-69]. Analyses of incipient phenotypic decoupling in other animals will help determine whether accelerated gene evolution only occurs after key innovations or fundamental shifts in life-history traits.

We next looked for evidence of processes that may have been lost or gained during phenotypic decoupling in eusocial evolution (Section 10 in Additional file 1; Additional file 5). We identified 431 caste-biased genes from across eusocial insect species that are also conserved in the 19 insect species genomes available. From this conserved set of genes, 12 GO terms were over-represented in highly eusocial insects (see Section 10 in Additional file 1 for species list) relative to *P. canadensis*, and 217 terms were under-represented (Section 10 in Additional file 1; Additional file 5). Under-represented groups (which include regulatory and signaling processes) may be less important in complex eusociality and hence not maintained by stabilizing selection. However, we cannot exclude the possibility that these differences are wasp-specific. Over-represented terms may be important in complex eusocial behavior [30]. These included processes previously highlighted for putative roles in caste determination (for example, oxidation reduction, metabolic processes) [39], suggesting they reflect the level of eusociality rather than differences in ecology or lineage.

## Conclusions

Our RNAseq analyses of phenotypic decoupling in *P. canadensis *provide some intriguing novel insights into the evolution of alternative phenotypes, and the early stages of caste evolution and sociality. First, they highlight the importance of novel genes in phenotypic diversity, emphasizing the need to expand existing genomic datasets beyond established model organisms to include a wider range of taxonomic groups. Secondly, they add important insights to the current emerging picture that much of the molecular changes that accompany social evolution occur predominantly in the worker rather than queen caste, with an over-representation of novel caste-biased genes in workers. These features may be important in facilitating the evolution of behavioral complexity in the worker caste. We show that these patterns are evident even at the early stages of sociality, where castes retain plasticity. Further, contrary to highly eusocial species, genes involved in caste differentiation do not appear to be subject to relaxed selection at the early stages of sociality. Diversity in the molecular regulation of castes across social lineages is expected if many pleiotropic genes involved with sexual conflict are lost in the early stages of social evolution, when ancestral monogamy is required to generate the conditions for the evolution of worker behavior [10]. Subsequent evolution of queen and worker behaviors, therefore, may take very different molecular pathways in different lineages, requiring new gene networks to evolve independently [28]. Further RNA-seq studies on other species (for example, [70]) and also genome sequencing to explore the potential role of alternative splicing (for example, [71]) will help determine the complementary roles of conserved and novel molecular processes in shaping social and other polyphenisms, bringing us closer to understanding how genomes give rise to phenotypic diversity in general.

## Materials and methods

### Source material

*P. canadensis *wasps of known behavioral repertoires were collected from wild populations in Panama, in July 2009 (Punta Galeta, Colon). All wasps were collected directly off their nests with forceps around midday during the active periods (that is, sunny weather) and preserved immediately in RNAlater (Ambion, Invitrogen, Applied Biosystems), and stored at -20°C until analysis (Section 1 in Additional file 1).

### Transcriptome sequencing, assembly and analyses

454 sequencing of pooled samples of 37 wasps across phenotypes (5 to 18 individuals per phenotype; 2.1 million reads, 80% brain, 10% abdomen, and 10% antennae) was used to generate a reference transcriptome (Section 2 in Additional file 1). Newbler v2.3 was used to generate the final assembled gene set (Table S1 in Additional file 1). Transcripts were annotated using GO categories assigned using BLASTx of GenBank NR databases with a conservative e-value threshold of 10^-5^, and Blast2Go was used to assess enrichment of GO terms among phenotypes (Section 4 in Additional file 1). Illumina sequencing of 14 biological replicates (>377 million reads) across 5 lanes was conducted to quantify differential gene expression, expressed as RPKM (reads per kilobase per million) values (Section 8 in Additional file 1). We trialed a number of methods for identifying differentially expressed genes and settled on a novel non-parametric method (NOISeq [59]) This method infers the noise distribution from the data and performs pairwise comparison of the samples to identify differentially expressed genes. A variety of probability thresholds were tested (Section 8 in Additional file 1). For the GO analysis we used a q-value >0.6 that represents a 50% chance that the gene is differentially expressed rather than not differentially expressed.

### Phylogenetic analyses

Protein sequences were aligned using MUSCLE [72], with default parameters. This is a multiple sequence aligner that includes an iterative alignment refinement phase to overcome known pitfalls of the progressive alignment strategy. Subsequently, poorly aligned regions of the alignment were trimmed with trimAl v1.3. [73] to remove columns with gaps in more than 30% of the sequences. A maximum-likelihood analysis was conducted on the concatenated alignment containing 33,506 sites using PhyML v3.0 [74]. Out of a total of five evolutionary models (LG, JTT, WAG, VT, BLOSUM62), the general replacement model LG (after 'Lee and Gascuel') was found to be the best fitting model using the AIC criterion [75]. In all cases four categories of evolutionary rates were used, estimating the gamma shape parameter and the proportion of invariable sites from the data. Branch supports were obtained using an approximate likelihood ratio test as implemented in PhyML ('Minimum of SH and chi-square' option). The resulting topology was compared to an alternative topology placing *Polistes *as a sister group to the ants. To do so, the phylogeny was re-computed, fixing the monophyly of *Polistes *and ants, but allowing the rest of the topology to be optimized. Support for the two topologies were compared using a Shimodaira-Hasegawa test, as implemented in CONSEL [76]. A Bayesian analysis was conducted as implemented in PhyloBayes [77], using the default CAT model and running two independent MCMC runs during 300,000 generations, and sampling every 100 generations. Consensus trees were built after removing the first 20% sampled trees and using a majority consensus rule.

### Data access

Raw sequence data are available at the European Read Archive (accession number ERP001342). The Transcriptome Shotgun Assembly project has been deposited at DDBJ/EMBL/GenBank under the accession GAFR01000001-GAFR01045087. The version described in this paper is the first version, GAFR01000000. All data and datasets can also be accessed at [78].

## Abbreviations

bp: base pair; GO: Gene Ontology; lncRNA: long non-coding RNA; NR: non-redundant; ORF: open reading frame; RPKM: reads per kilobase per million.

## Competing interests

The authors declare that they have no competing interests.

## Authors' contributions

SS conceived the idea and designed the research. SP and SS performed the fieldwork, SP performed wet-lab work. RC and Ff-C conducted the MIRA assembly; PGF and RG designed and conducted all other bioinformatics analyses. TG conducted the gene evolution and phylogenetic analyses. SS and PGF wrote the paper, with comments from all authors. All authors read and approved the final manuscript.

## Supplementary Material

Additional file 1 **Sections 1 to 10 and Tables S1 and S2**. Additional methods and results referred to in the main text can be found here. Table S1: comparison of transcriptome assemblies. Number of transcripts, genes and respective transcript length statistics for the optimal assemblies generated with Version 2.3 and Version 2.5 of GS Newbler Assembler, and Version 3.0.5 of Mira Assembler and Oases (with Illumina paired-end sequence data). Table S2: numbers of differentially expressed (q > 0.6) genes and transcripts was robust to different numbers of biological replicates. Click here for file

Additional file 2 **Read coverage from the 454 transcriptome assembly**. **(A) **Distribution of the number of 454 reads per transcript (truncated to transcripts with less than 500 reads). **(B) **Distribution weighted by the transcript length (truncated plot). The majority of transcripts had more than 5.19 reads, on average. Click here for file

Additional file 3 **Functional groups identified in the *P. canadensis *transcriptome**. Distribution of the number of genes according to different GO terms from the 454 pooled transcriptome assembly. Categories were filtered by a minimum number of sequences: 50 for cellular component; 200 for biological process; and 100 for molecular function. Click here for file

Additional file 4 **Information on the best BLAST hits in the NR database for each transcript; noncoding RNA potential for genes with and without hits in NR**. Tabs: 'besthits_with_NR', information extracted from the best hits for each transcript; 'PortraitScore_transcriptsWithNOHits', 'PortraitScore_transcriptsWithHits', score from the portrait program. Click here for file

Additional file 5 **Information on functional analyses and comparison with existing caste expression data from other species**. Tabs are as follows. 'Toth2010_Pmetricus_Pcan', mappings between *P. metricus *and *P. canadensis *transcripts and their respective expression levels and whether they are significantly differentially expressed in *P. canadensis*. 'Whitfield2003_50cDNAS', mappings between 50 most discriminative cDNAs for worker behaviors in *A. mellifera *from Whitfield *et al*. [18] and *P. canadensis*, with respective expression values. 'GO-Enrichment Differential Expressed (DE) Genes', list of over/under-represented GO terms for genes significantly up- and down-regulated in each caste comparison. Genes were categorized according to biological processes (P), molecular function (F) and cellular component (C). Most specific terms were retrieved with Blast2GO and correspond to the leaf nodes in the GO tree, this excludes cases where parent and child nodes may be included. Column labels are: Test (number of genes in the test set annotated with this GO term); Ref (number of genes in the ref set annotated with this GO term); notAnnotTest (number of genes in the test set NOT annotated with this GO term); notAnnotRef (number of genes in the reference set NOT annotated with this GO term), where 'test' set is the set of genes up-regulated in queens, and the 'ref' set those up-regulated genes in workers, and vice-versa. 'GO-Enrichment', list of over/under-represented GO terms for the *P. canandensis *genes that are conserved across the genomes of 6 eusocial insects. 'genes_with_Behavior_GOterm', genes classified with GO term thought to be relevant to behavior, their respective descriptions and direction of differential expression in *P. canadensis*. 'genes_accelerated_Pol_Bee_Ants', genes found accelerated in *Polistes*, bee and ants, and direction of differential expression in *P. canadensis*. 'genes_accelerated_polistes', genes found accelerated only in *Polistes*; and direction of differential expression in *P. canadensis*. 'phylogenetic marker identifiers', a list of the 93 marker genes used in the phylogenetic analyses. These correspond to phylomeDB codes in the Aphid phylome used as the reference markers. Sequences for these identifiers can be obtained from Huerta-Cepas *et al*. [57]. Click here for file

Additional file 6 **Number of mapped reads per individual**. Click here for file

Additional file 7 **Basic information about the assembled transcripts**. Tabs: 'transcript_length', length of each transcript; 'transcript_to_gene', correspondence between transcript and gene. Click here for file

Additional file 8 **Cumulative distribution of gene expression for the four phenotypes**. Click here for file

Additional file 9 **Expression values in RPKM (reads per kilobase per million) for all the individuals, indicating which caste each isotig is up-regulated in**. Click here for file

Additional file 10 **NOISeq probability values for each transcript for the comparison of each caste (that is, W (worker), Fo (foundress), Q (queen), or C (callow)) versus the others, and between queens and workers**. Expression values for each caste and comparison, differential expression statistics ('M' and 'D' [12]), probability of differential expression ('prob'); 'ranking', which is a summary statistic of 'M' and 'D'. Click here for file
