# Supplementary material for: Transcriptome analyses of primitively eusocial wasps reveal novel insights into the evolution of sociality and the origin of alternative phenotypes
Source: Genome Biol. 2013 Feb 26;14(2):R20. doi: 10.1186/gb-2013-14-2-r20 (PMC4053794; doi:10.1186/gb-2013-14-2-r20)
Supplement: Additional file 1 — Sections 1 to 10 and Tables S1 and S2. Additional methods and results referred to in the main text can be found here. Table S1: comparison of transcriptome assemblies. Number of transcripts, genes and respective transcript length statistics for the optimal assemblies generated with Version 2.3 and Version 2.5 of GS Newbler Assembler, and Version 3.0.5 of Mira Assembler and Oases (with Illumina paired-end sequence data). Table S2: numbers of differentially expressed (q > 0.6) genes and transcripts was robust to different numbers of biological replicates. [file gb-2013-14-2-r20-S1.DOCX]

**Additional File 1**

**Transcriptome analyses of primitively eusocial wasps reveal novel insights into the evolution of sociality and the origin of alternative phenotypes**

Pedro G Ferreira*^1,6^*, Solenn Patalano*^2,3^*, Ritika Chauhan*^4^*, Richard Ffrench-Constant*^4^*, Toni Gabaldón*^1^*, Roderic Guigó*^1^* , Seirian Sumner*^2,5*^*

*^1^Centre for Genomic Regulation (CRG-UPF), Doctor Aiguader, 88, 08003 Barcelona, Spain.*

*^2^Institute of Zoology, Zoological Society of London, Regent’s Park, NW1 4RY, UK*

*^3^The Babraham Institute, Babraham Research Campus, Cambridge, CB22 3AT, UK*

*^4^ Centre for Ecology and Conservation, Biosciences, University of Exeter, Tremough, Penryn, TR10 9EZ, UK*

*^5^ School of Biological Sciences, University of Bristol, Woodland Road, Bristol, BS8 1UG, UK*

*^6^ Current address: Department of Genetic Medicine and Development, University of Geneva Medical School, 25 Rue Michel Servet 1, 1211 Geneva, Switzerland.*

** Corresponding author:* Seirian.Sumner@Bristol.ac.uk

This document contains additional information (Sections 1-10) on Methods, Results and Additional Tables referred to in the main text.

**1) Sample collection.** A total of 37 individuals were used to generate the 454 representation of transcription in adult females*. a) 454 samples:* Queens (n=5) were established dominants and were collected within 24 hours of being seen egg-laying. Mature ovarian development was further confirmed from dissections where all queens had mature eggs (>2mm) and sperm in their spermathecae [[1](#_ENREF_1)]. Workers (n=5) were of a range of ages and were collected as they returned to the nest with solid forage material (e.g. caterpillar prey) which was then fed to the brood. None of the workers had developed ovaries. Ovarian dissections of callows (n=9) confirmed they were freshly emerged, through the presence of meconium (the [metabolic](http://en.wikipedia.org/wiki/Metabolic) waste products generated by the pupa, which are expelled after [eclosion](http://en.wikipedia.org/wiki/Pupa)) and no ovarian development. Foundresses (n=18) were a mix of subordinate and dominant individuals, with a range of ovarian development. We pooled many foundresses because we wanted to be sure to capture the range of behaviors and social ranks existing in these large, socially diverse groups.

*b) Illumina samples:* A total of 14 biological replicates were used for Illumina short-read sequencing. Individuals were chosen carefully to represent the full behavioral diversity of each phenotype. Queens and workers were obtained from different colonies to avoid any gene expression bias due to genotype or colony-specific attributes. All queens (n=4) had been observed as the primary egg-layer on their established nest, had mature eggs (>2mm long) in their ovaries and were inseminated. All workers (n=6) were observed returning to their nests with solid forage material (e.g. caterpillar). Two workers had a small amount of ovarian development but no mature eggs (egg length <1mm), whilst the other workers had no ovarian development at all. Half of the workers were mated, as is typical in this species (1). An additional pool of 6 workers was also used, collected from different nests, all of which were observed foraging. The foundresses (n=2) were collected from the same foundress nest so that we could be sure of relative dominance. The egglaying foundress (Fo_34_Dom) was identified from censuses conducted over several days prior to collection as the only female to sit directly on the nest; she had well developed ovaries with many mature eggs. The other foundress was her subordinate (Fo_34_Codom), who always sat off the nest and showed less ovarian development, having only 3 pre-mature eggs (egg length = 1.6mm) and no development higher up the ovarioles. Foundresses were mated, and they had originated from different post-emergence nests indicating that they were not sisters. Callows (n=2) were newly emerged, as shown by their black eyes and the concurrence of hatched pupal cells on their nest. Neither had any ovarian development nor were they mated. However, callow C_109 had meconium in her abdomen, whilst callow C_108 did not, indicating the former female was freshly emerged (<12 hours) whilst the latter was likely to be 1-2 days old.

**2) Transcriptome sequencing and assembly.** Total RNA was extracted from brains, antennae and abdomens across all phenotypes (Figure 1, main text) using Trizol reagent (Invitrogen) following standard protocols. Transcription in the brain informs about behavior and is the region where most transcripts in the genome are expressed. The addition of abdominal and antennal tissue enhances transcript discovery for reproductive and sensory-related processes, providing a more holistic resource for subsequent sociogenomic studies. RNA was DNAse treated (Turbo, Promega), purified (Nucleospin) and checked for RNA quality and integrity on an Agilent Bioanalyser before pooling within phenotypes in the following proportions: 80% brain, 10% abdomen, and 10% antennae. Normalised cDNA libraries were prepared from 5µg of RNA using MINT-universal cDNA synthesis kits (Evrogen, Russia). Libraries were normalised in order to ensure all transcripts were represented in the reference transcriptome. Roche 454 sequencing libraries were prepared from 5µg of each normalised caste-specific cDNA and sequenced on a Roche 454 Life Sciences GS FLX Titatnium Series sequencer (GenePool, University of Edinburgh). The four pooled samples were sequenced separately, and standard on-instrument base-calling and quality screening was performed, generating 2.1 Million 300bp (average) reads (600MB of sequence).

Results can be influenced by the way in which the data is assembled into transcriptomes, especially for *de novo* assembly of non-model organisms as in our study [[2](#_ENREF_2)]. In order to select the best assembly for our data we used two versions of the Roche GS Assembler (Newbler - v2.3 and v2.5), and another assembler, Mira v3.0.5. We then compared the number and the length of the transcripts for the two assemblies. For the Mira assembly *de novo* normal EST 454 parameters were used and assembled using the semi-automated pipeline *est2assembly* [[3](#_ENREF_3)]. For the Newbler assemblies the programs were run with the cDNA assembly option. This program uses the nomenclature of *isotig* to refer to a putative individual transcript and the term *isogroup* for a gene or locus. Each isogroup consists of a set of contigs connected in a network graph. A path through this graph yields an isotig and multiple paths indicate possible splice-variants; thus an isogroup may have one or more isotigs. For the rest of this article we use the term *transcript* to refer to an isotig and *gene* for an isogroup. For each version of the program, the best assembly was selected by generating iteratively a set of assemblies until an optimal combination of the input parameters was found. The Mira assembly produced many short reads and many more transcripts than both Newbler assemblies, suggesting a lower quality and less stringent assembly (Table S1). The results and conclusions were very similar for the two Newbler assemblies. However, Newbler v2.3 generated more transcripts and longer ones and so we chose to use the v2.3 assembly for the rest of the study.

To confirm equal representation of all castes in our transcriptome dataset, we constructed a total of five assemblies: four caste–specific transcriptomes, and a pooled one (henceforth called ‘Reference’), consisting of all the reads in the four datasets. We used the cDNA assembly option in Assembler v2.3, with a minimum read length of 40bp. Optimal assemblies were selected by varying input parameters, and the assembly providing the highest N50 value and the highest number of assembled reads was selected. The optimal parameters were a minimum overlap length of 40 nucleotides and a minimum overlap identity between reads of 90%, resulting in an N50 of 2111. In total, from the 2,676,822 reads (length median: 306; mean:292; max:1196), 1,538,949 (74.4%) reads were fully assembled and 193,059 (9.3%) partially assembled (Dataset S1: ‘transcripts’). The Reference assembly yielded 45,326 transcripts (mean length = 1505 bp; longest transcript = 11,893 bp), which assembled into 26,284 genes of which 22.3% had more than 1 transcript. Individual castes contributed 37% to 39% of transcription activity, confirming equal representation of each caste in the Reference. The Reference assembly was used for all subsequent analyses. For comparison purposes we also performed an assembly with Illumina paired-end sequencing data, using the Oases transcriptome assembler program [[4](#_ENREF_4)]. It resulted in a higher number of assembled transcripts with shorter length (see Table S1), indicating higher levels of gene fragmentation, and thus lower quality, than the 454 assembly.

**3) Completeness of assembly.** To determine how complete our assembly was we scrutinised it in four different ways: (1) read coverage; (2) completeness of the gene space; (3) comparison with existing EST data; (4) transcript distribution across genomes in related species. Firstly, we calculated the distribution of read coverage across all transcripts (see Additional File 2). There was an average of 125 reads per transcript (standard deviation = 179). Across all transcripts, 99.8% had more than one read and 93.7% had more than 5 reads, indicating high read coverage across almost all transcripts. Secondly, we assessed coverage of gene space by looking for representation of core eukaryotic genes (CEGs) [[5](#_ENREF_5)]. CEGs are a set of genes expected to be present in all complete genome sequences of higher eukaryotes. The CEG set corresponds to 248 proteins derived from six model organisms: *Homo sapiens*, *Drosophila melanogaster*, *Arabidopsis thaliana*, *Caenorhabditis elegans*, *Saccharomyces cerevisiae* and *Schizosaccharomyces pombe*. In our transcriptome assembly we detected (e-value < 10^-6^) 246 and 1464 hits in *D. melanogaster* and the 6-model organisms representing 99.2% and 98.4% of the CEG set respectively. This indicated almost complete representation across the gene space. Thirdly, we mapped the assembled sequences against existing Sanger sequences for our study species [[6](#_ENREF_6)]. Forty three ESTs are available for *P. canadensis* in the NCBI Dataset, and 36 of these were present in our transcriptome assemblies. In each case, the sequences were identical (e-value < 10^-6^). These high values provide a good indication that sequencing was accurate, and that the assembly covers the majority of the transcriptome space of *P. canadensis*. The 7 undetected ESTs are likely to be specific to body tissues not represented in our transcriptome, since the published ESTs in Sumner et al 2006 were derived from cDNA prepared from whole bodies, rather than predominantly brain as in this study. Finally, we examined putative distribution of transcripts across chromosomes using the honeybee *Apis mellifera* as a scaffold [[7](#_ENREF_7)]. All transcripts defined in *P. canadensis* were blasted against the Ensembl 2010 *A. mellifera* dataset which corresponds to 27,755 annotated genes and transcripts [[8](#_ENREF_8)]. Of the *A. mellifera* transcripts, 48% (13,432) had significant hits (e-value< 10^-4^) with *P. canadensis,* and these transcripts were distributed equitably across the honeybee chromosomes. In addition, we compared the level of overlap of *A. mellifera* and *P. canadensis* with the genomes of all other Hymenopterans with sequenced genomes to date (Table 1 main text). These four analyses indicate strongly that our pooled assembly is a comprehensive and unbiased representation of transcription across the genome as a whole, and provides a comprehensive reference transcriptome for the behaviors exhibited by colony-level individuality in *P. canadensis*.

**4) Functional annotation of the transcriptome.** The Blast2Go program extracts Gene Ontology (GO) terms from the results of Blast searches and provides GO annotation for each query sequence. This can then be used to visualize the results and perform statistical analyses, in particular enrichment analyses of GO terms. To annotate the transcripts, a BLASTx against the Genbank non-redundant (NR) protein dataset was performed with a relatively conservative e-value threshold of 10^-5^. For each transcript the best hit was selected. 49.4% and 37.4% of the transcripts and genes respectively, have significant matches with the NR Dataset (Additional File 4: ‘annotation’). Half (50.6%) of the transcripts did not have a significant hit: these are likely to represent novel transcripts that have not been sequenced before, but we cannot rule out that these may be incompletely assembled sequences. 16.9% of the annotated transcripts correspond to genes with more than one annotated transcript. The function of each gene is assigned based on the annotation of the respective transcript with the best hit (lower e-value). We explored the distribution across other species of the best BLAST hits. We were able to assign GO annotations to 15,623 transcripts and 6663 genes. A total of 2242 distinct gene ontology (general GO) terms were assigned, with an average of 7.3 terms per gene. Genes were categorized according to biological processes (P), molecular function (F) and cellular component (C) (Additional Files 3-5).

**5) Identifying putative non-coding RNAs. a) Protein coding potential.** We used PORTRAIT [[9](#_ENREF_9)] to characterize the protein coding potential of those transcripts in the reference transcriptome for which no similarity to NR protein coding genes was found. This program was developed for ncRNA analyses of transcriptomes from poorly characterized species, with low quality or incomplete coverage of the assembled transcripts. It uses a classification model based on a set of non-redundant protein sequences from the three most comprehensive ncRNA Datasets. For each sequence a score between 0 and 1 is provided as a classification score for protein-coding (PC, > 0.5) or non-coding (NC, < 0.5). **b) Open reading frame**. Translation to protein sequence and open reading frames were calculated with the EMBOSS tools. **c)** **Homology to protein domains**. Transcripts were translated into protein coding sequences, and scanned against a profile-HMM database using default parameters (HMMscan). We detected known domains for 13,153 transcripts (6398 genes), and no BLAST hit or domain for 20,650 transcripts (15,765 genes). We then categorized the sequences as potential non-protein coding by applying a three step filtering: i) determining how many isotigs are longer than 300 bps and have an ORF shorter than 90aa (96% of the Amel transcripts have an ORF greater than 90aa); ii) imposing a PORTRAIT score lower than 0.5; iii) those lacking homology with known protein domains as determined by HMMscan.

**6) Effects of transcriptome-based gene assembly on gene characterisation and annotation.**  To assess whether the fragmentation level in our transcriptome may falsely elevate the total number of isogroups, we used mapping information from the Illumina paired end reads (230bp of average insert size). For each sample we selected the reads where both ends map uniquely in the transcriptome. We counted the number of times that paired end reads map simultaneously to two different isogroups (with a minimum of 5 reads per sample); i.e. represent single transcripts that span two ‘genes’. We detected 1049 of such cases, with 740 expressed in more than one sample. Thus, out of a total of 22,460 putative protein coding isogroups ~ 1000 may be artifacts arising from a fragmented assembly and therefore have a merged assembly with other isogroups.

Additionally, we looked for genes in the OGS for *A. mellifera* which have 2 or more non-overlapping hits from different *P. canadensis* isogroups. Using an e-value of <10^-5^, we identified 750 *A. mellifera* genes corresponding to 871 unique pairs in *P. canadensis*. 143 of these pairs of isogroups are common to the 1049 unique pairs derived from the paired end mapping. From these analyses we obtained a set of 1777 unique pairs, representing ~ 1800 isogroups that may not be completely assembled and can possibly be merged.

In 65% (1156) of the unique isogroup pairs both isogroups have a hit in NR databases, and for 15% (270) neither isogroups have a match. In the remaining 20% only one of the isogroups have a hit. These values seem to indicate that only a relatively small fraction of the novel genes can be explained by an incomplete assembly, since the majority (80%) of the isogroup pairs have no hit or a known hit in both of the isogroups.

**7) Illumina sequencing of individuals and mapping to transcriptome.** In order to obtain a first look at transcriptional decoupling between queens and workers, we sequenced RNA from brains of individual wasps from each caste using the Illumina short-read platform (samples described in Section 1 above). This method is highly sensitive and allows quantification of gene expression through counts of the gene-specific transcripts mapped to the 454 reference transcriptome. RNA was prepared in the same way as for 454 sequencing (see Section 2 above). mRNAs were purified from total RNA, fragmented, and reverse-transcribed into cDNA. Then, cDNA was processed using the mRNA-Seq Sample Preparation Kit (Illumina) to generate an Illumina library. Following quality checks, the library was denatured and loaded onto an Illumina Flowcell. Sequencing was conducted through sample multiplexing. Each sample was labelled with a unique identifier, samples were pooled and then run in a five sequencing lanes of an Illumina Genome Analyzer II. This strategy provides an extremely high-throughput, cost-effective assay of global transcript abundance in several independent samples. Reads were 40 and 46 base-pairs long

We obtained a total of 397M reads (159.5M for workers, 119.4M for queens, 57.4M for foundresses and 60.4M for callows). Reads were mapped to the 454 Pool transcriptome using GEM mapper [[10](#_ENREF_10)], allowing two mismatches in the mapping (Additional Files 6 & 7). The paired-end parts of the reads were treated independently. 99.8% of the 26,284 genes in the reference transcriptome were detected in the pooled Illumina dataset, attesting again to the completeness of our reference transcriptome. The remaining genes are likely to derive from the non-brain tissue in the 454 sample.

**8)** **Quantifying transcript abundance and differential gene expression** a) *Transcript and gene quantification.* After mapping, we calculated the expression value for each transcript. This was done using the Reads Per Kilobase per Million (RPKM) measure, which was designed to allow for direct comparison between different experiments [[11](#_ENREF_11)]. The formula was: RPKM_i_ = C_i_ / (L_i_ X N) X 10^9^

This takes into account the number of reads mapped in the transcript *(C),* the length of the transcript *(L),* and the total number of mapped reads in the experiment *(N).* All mapped reads were used for this calculation. We use a weighted scheme where reads mapped uniquely have a higher weight than reads with multiple mapping positions. The count of reads per transcript can be written as *C_i_ = ∑_j_^M^ 1/m_j_,* where *M* is the number of mapped reads and *m_j_* the number of times that read *j* maps in the transcriptome. The expression of each gene was calculated as the average of the RPKM of all the transcripts associated to that gene (see Additional File 8 for the distribution of expression values).

For the differential expression analysis we used a novel method called NOISeq [[12](#_ENREF_12)]. This is a non-parametric method that empirically models the noise in the data. The method estimates the noise distribution by performing pairwise comparison between all the samples in the tested conditions, making it suitable to compare conditions with different sample sizes. It then estimates the probability of a gene being differentially expressed. Evaluation shows that the method is robust against different levels of sequencing depth and is stable across a number of variable conditions. Comparisons with other statistical methods for gene expression analyses, in datasets where RT-PCR controls for gene expression are available, shows that the method has a better precision/recall and much lower false discovery rate [[12](#_ENREF_12)]. Since the method shows equal performance for read-counts and RPKM values we used the latter. NOISeq reports for each gene a probability of this gene being differentially expressed between the two conditions, expressed as a q value between 0 and 1. We report differentially expressed genes for three thresholds: q=0.75, q=0.67 and q=0.6, corresponding respectively to an odds of 200%, 100%, and 50% more likely for the gene to be differentially expressed than non-differentially expressed (Additional File 10). As many of the genes are not annotated we used the more inclusive threshold of q=0.6 for the GO analysis. This method was also robust to comparison with different number of biological replicates (Table S2). No significant difference was observed between the length of differentially and non-differentially expressed genes.

**9) Effects of sequence fragmentation on differential expression.** RNAseq produces very short sequences that are then aligned into contigs that represent individual transcripts. There is a chance that two reads belonging to the same transcript could be wrongly assigned to two separate transcripts. Such fragmentation may affect our analysis of differential expression. To determine this, we explored the distribution of the fragmented isogroups among the differentially expressed isogroups. For 134 of the 1777 unique isogroups pairs (see above) both isogroups were present in the 2442 differentially expressed isogroups. This means that these may be fragmented and the number of differentially expressed isogroups may be 67 (2.74%) less. There are also 158 cases in the 1777 unique pairs, where one of the isogroups of the pairs is differentially expressed (6.47%). This means that either the other part is not differentially expressed, or that the pairs that we are finding are not really connecting two isogroups. In summary, the overlap with the putative fragmented genes shows that only a relatively small fraction (less than 9.21%) of the differentially expressed genes may be affected. Therefore, gene fragmentation does not have a substantial impact in our conclusions.

**10) Molecular evolution.** Orthologs of *Polistes* genes were predicted by Best-reciprocal blast hits (e-value < 10^-5^) in each of the other hymenopteran species available. Protein sequences of orthologous groups were aligned using MUSCLE [[13](#_ENREF_13)] with default parameters. TrimAl [[14](#_ENREF_14)] was used to trim all gaps in the alignment and back translate them into their codon sequences, using information from the respective CDS. The program CodeML from the PAML package [[15](#_ENREF_15)] was used to fit the alignment data to branch models of codon evolution by Maximum Likelihood to identify differences in selective pressures (w) within the tree. For each alignment the likelihoods of three models of evolution were compared by using a Likelihood Ratio Test (LRT). These models consisted of H0 (neutral model): there are no significant differences in evolutionary ratios across all the lineages considered; H1: the gene has a specific w in the *Polistes* lineage, higher than a common w in the rest of the tree; H2: the gene has a higher and similar w in the three lineages leading to sociality (*Polistes, Apis,* Ants); H3: within *P. canadensis* queen- and worker-biased genes differ in their evolutionary rates. Lineages with dS>2.5 in the relevant lineages were considered saturated and discarded. The scenario in Figure 3a, main text was used. We found no evidence that caste-biased genes were more likely to have accelerated rates in the tested lineages than non-caste-biased genes, mainly because only one of the caste-biased genes showed accelerated rates in each case (1/34 (H1) and 1/141 (H2)). Similarly, although the differences were greater, no significant differences in lineage-specific acceleration of evolutionary rates were found between worker-biased (4/248) and queen-biased genes (1/187; p=0.56) (Additional File 5). Similar results were obtained (>95% of agreement in all cases), when using an alternative topology maintaining the monophyly of *Vespoidea (*i.e Figure 3b main text).

We examined gene conservation of the caste-biased genes in *P. canadensis* across 19 species of insects whose genomes have been sequenced, which included 6 highly eusocial insect species (*Apis mellifera, Atta cephalotes, Camponotus floridanus, Harpegnathus saltator, Linepithema humile, Pogonomyrmex barbatus*) and 13 non-social species (*Aedes aegypti, Anopheles gambiae, Acyrthosiphon pisum, Bombyx mori, Culex pipiens, Droshophila melanogaster, Daphnia pulex, Ixodes scapularis, Nasonia giraulti, Nasonia longicornis, Nasonia vitripennis, Pediculus humanus, Tribolium castaneum)*. In total, 13,126 *P. canadensis* genes had significant hits across all 19 species. To identify rapidly evolving genes we used Likelihood Ratio Tests to compare rates of gene evolution between lineages, using the phylogeny generated from our study. Finally, we compared caste-biased gene conservation within and between social lineages by looking for overlap between the caste-biased genes we identified in our study with those identified in *Apis mellifera* and *Polistes metricus* (Additional File 5).

**Additional Database files and Data Access**

Raw sequence data is available at the European Read Archive (accession number: ERP001342). The Transcriptome Shotgun Assembly project has been deposited at DDBJ/EMBL/GenBank under the accession GAFR01000001-GAFR01045087. The version described in this paper is the first version, GAFR01000000. All data and datasets can also be accessed at: <http://genome.crg.es/~pferreira/pcandata/pcan.htm>

**References**

1. Sumner S, Kelstrup H, Fanelli D: **Reproductive constraints, direct fitness and indirect fitness explain helping behavior in the primitively eusocial wasp, Polistes canadensis.** *Proc R Soc Lond B* 2010, **277:**1721-1728.

2. Kumar S, Blaxter M: **Comparing de novo assemblers for 454 transcriptome data.** *BMC Genomics* 2010, **11:**571.

3. Papanicolaou A, Remo S, Ffrench-Constant RH, Heckel DG: **Next generation transcriptomes for next generation genomes using est2assembly.** *BMC Bioinformatics* 2009, **10:**447.

4. Schulz MH, Zerbino DR, Vingron M, Birney E: **Oases: robust de novo RNA-seq assembly across the dynamic range of expression levels.** *Bioinformatics* 2012, **28:**1086-1092.

5. Parra G, Bradnam K, Ning Z, Keane T, Korf I: **Assessing the gene space in draft genomes.** *Nucleic Acids Res* 2009, **37:**289-297.

6. Sumner S, Pereboom JJM, Jordan WC: **Differential gene expression and phenotypic plasticity in behavioral castes of the primitively eusocial wasp, Polistes canadensis.** *Proc R Soc Lond B* 2006, **273:**19-26.

7. The Honeybee Genome Sequencing Consortium: **Insights into eusocial insects from the genome of the honeybee *Apis mellifera*.** *Nature* 2006, **443:**931-949.

8. Birney E, Andrews TD, Bevan P, Caccamo M, Chen Y, Clarke L, Coates G, Cuff J, Curwen V, Cutts T, Down T, Eyras E, Fernandez-Suarez XM, Gane P, Gibbins B, Gilbert J, Hammond M, Hotz HR, Iyer V, Jekosch K, Kahari A, Kasprzyk A, Keefe D, Keenan S, Lehvaslaiho H, McVicker G, Melsopp C, Meidl P, Mongin E, Pettett R, et al: **An overview of Ensembl.** *Genome Res* 2004, **14:**925-928.

9. Arrial RT, Togawa RC, Brigido Mde M: **Screening non-coding RNAs in transcriptomes from neglected species using PORTRAIT: case study of the pathogenic fungus Paracoccidioides brasiliensis.** *BMC Bioinformatics* 2009, **10:**239.

10. Marco-Sola S, Sammeth M, Guigo R, Ribeca P: **The GEM mapper: fast, accurate and versatile alignment by filtration.** *Nat Methods* 2012, **advance online publication**.

11. Mortazavi A, Williams BA, McCue K, Schaeffer L, Wold B: **Mapping and quantifying mammalian transcriptomes by RNA-Seq.** *Nat Methods* 2008, **5:**621-628.

12. Tarazona S, Garcıa-Alcalde F, Dopazo J, Ferrer A, Conesa A: **Differential expression in RNA-seq: A matter of depth.** *Genome Res* 2012, **21:**2213-2223.

13. Edgar RC: **MUSCLE: a multiple sequence alignment method with reduced time and space complexity.** *BMC Bioinformatics* 2004, **5:**113.

14. Capella-Gutierrez S, Silla-Martinez JM, Gabaldon T: **trimAl: a tool for automated alignment trimming in large-scale phylogenetic analyses.** *Bioinformatics* 2009, **25:**1972-1973.

15. Yang Z: **PAML 4: phylogenetic analysis by maximum likelihood.** *Mol Biol Evol* 2007, **24:**1586-1591.

| Assembler | Transcripts | Genes | Transcripts length > 500bp | TranscriptMedian Length | Transcript Avg. Length | Transcript N50 |
| --- | --- | --- | --- | --- | --- | --- |
| **Newbler V2.3** | **45 326** | **26 284** | **39 183** | **1098** | **1501** | **2111** |
| Newbler V2.5 | 43 261 | 26 630 | 37 127 | 1050 | 1457 | 2042 |
| Mira V3.0.5 | 43107 | - | 12,935 | 770 | 709 | 1803 |
| Oases (Illumina) | 76839 | - | 41117 | 549 | 1057 | 540 |

**Table S1 Comparison of transcriptome assemblies.**

Number of transcripts, genes and respective transcript length statistics for the optimal assemblies generated with Version 2.3 and Version 2.5 of GS Newbler Assembler, and Version 3.0.5 of Mira Assembler, and Oases (using Illumina paired-end sequence data).

**Table S2: Numbers of differentially expressed (q>0.6) genes and transcripts were robust to different numbers of biological replicates.**

| Comparison | UP  Genes(transcripts) | DOWN  Genes(transcripts) |
| --- | --- | --- |
| Worker (n=6) vs All Others | 2222 (2924) | 84 (109) |
| Worker (n=2) vs All Others | 2289 (3107) | 139 (194) |
| Queen (n=4) vs All Others | 47 (67) | 402 (522) |
| Queen (n=2) vs All Others | 186 (264) | 78 (95) |
| Foundress (n=2) vs All Others | 11 (15) | 2389 (3340) |
| Foundress (n=2) vs Others (n=2/phenotype) | 17 (23) | 1999 (2915) |
| Callow (n=2) vs All Others | 163 (238) | 1454 (1779) |
| Callow (n=2) vs Others (n=2 per phenotype) | 109 (139) | 516 (637) |
