# Supplementary figures and images for: Transcriptome analyses of primitively eusocial wasps reveal novel insights into the evolution of sociality and the origin of alternative phenotypes
Source: Genome Biol. 2013 Feb 26;14(2):R20. doi: 10.1186/gb-2013-14-2-r20 (PMC4053794; doi:10.1186/gb-2013-14-2-r20)

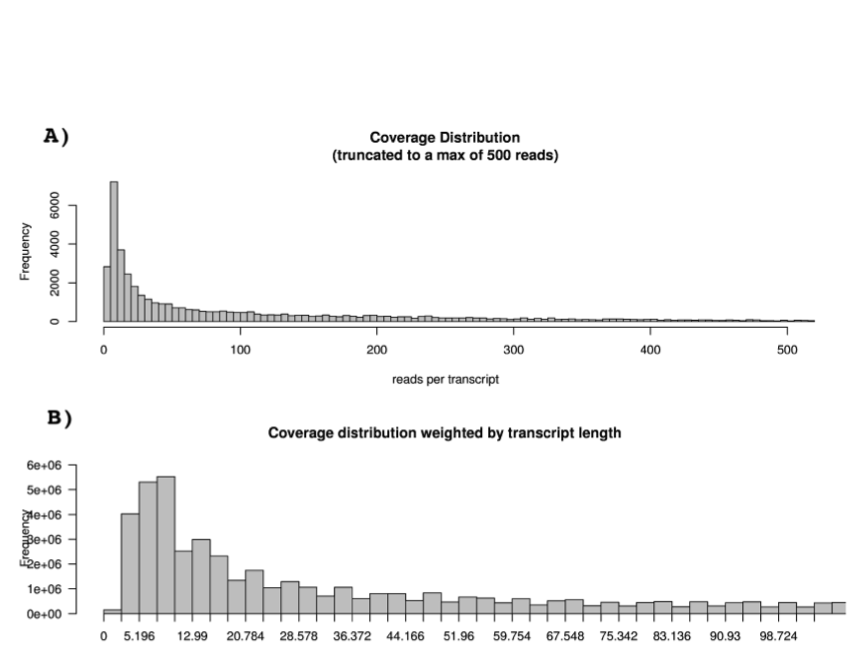

Supplement: Additional file 2 — Read coverage from the 454 transcriptome assembly. (A) Distribution of the number of 454 reads per transcript (truncated to transcripts with less than 500 reads). (B) Distribution weighted by the transcript length (truncated plot). The majority of transcripts had more than 5.19 reads, on average. [file gb-2013-14-2-r20-S2.TIFF]

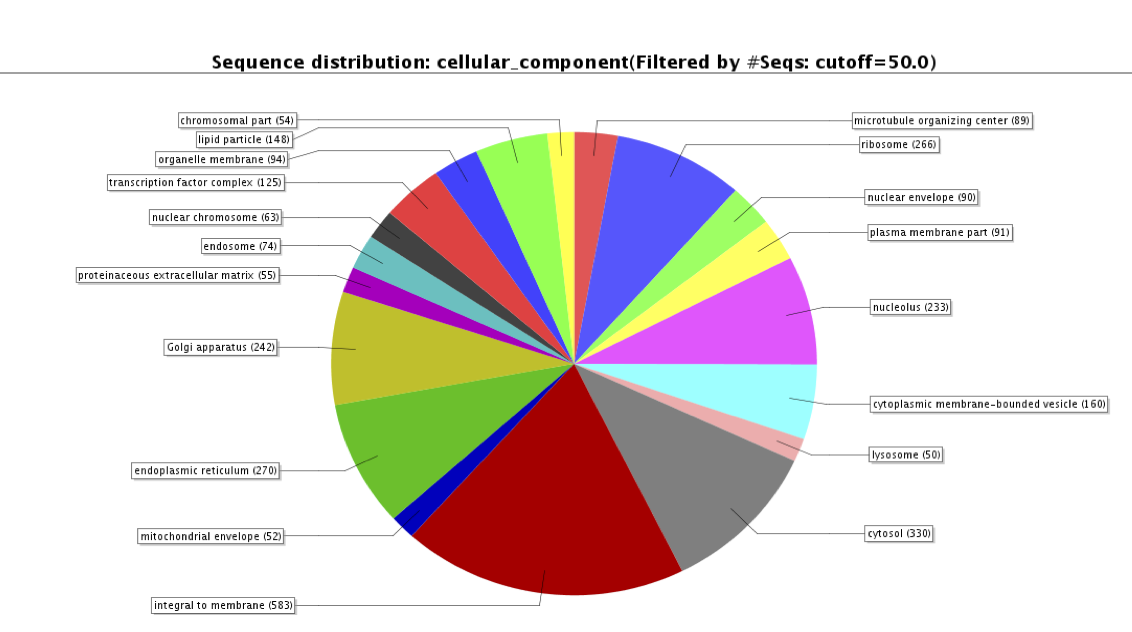

Supplement: Additional file 3 — Functional groups identified in the P. canadensis transcriptome. Distribution of the number of genes according to different GO terms from the 454 pooled transcriptome assembly. Categories were filtered by a minimum number of sequences: 50 for cellular component; 200 for biological process; and 100 for molecular function. [file gb-2013-14-2-r20-S3.TIFF]

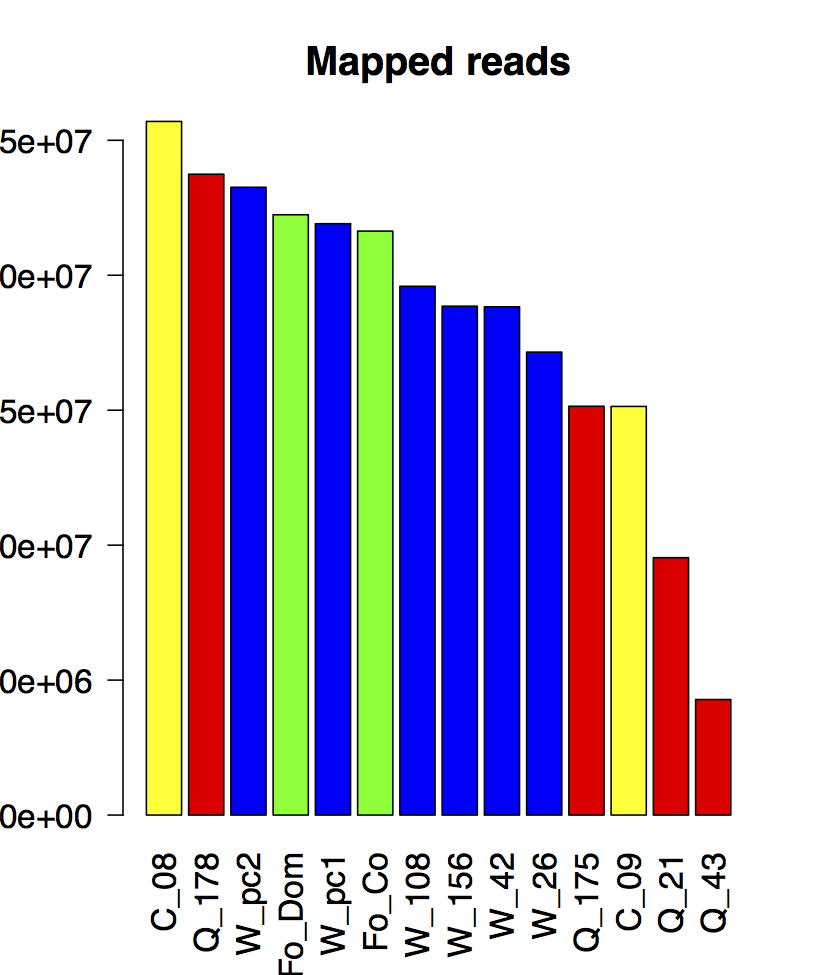

Supplement: Additional file 6 — Number of mapped reads per individual. [file gb-2013-14-2-r20-S6.TIFF]

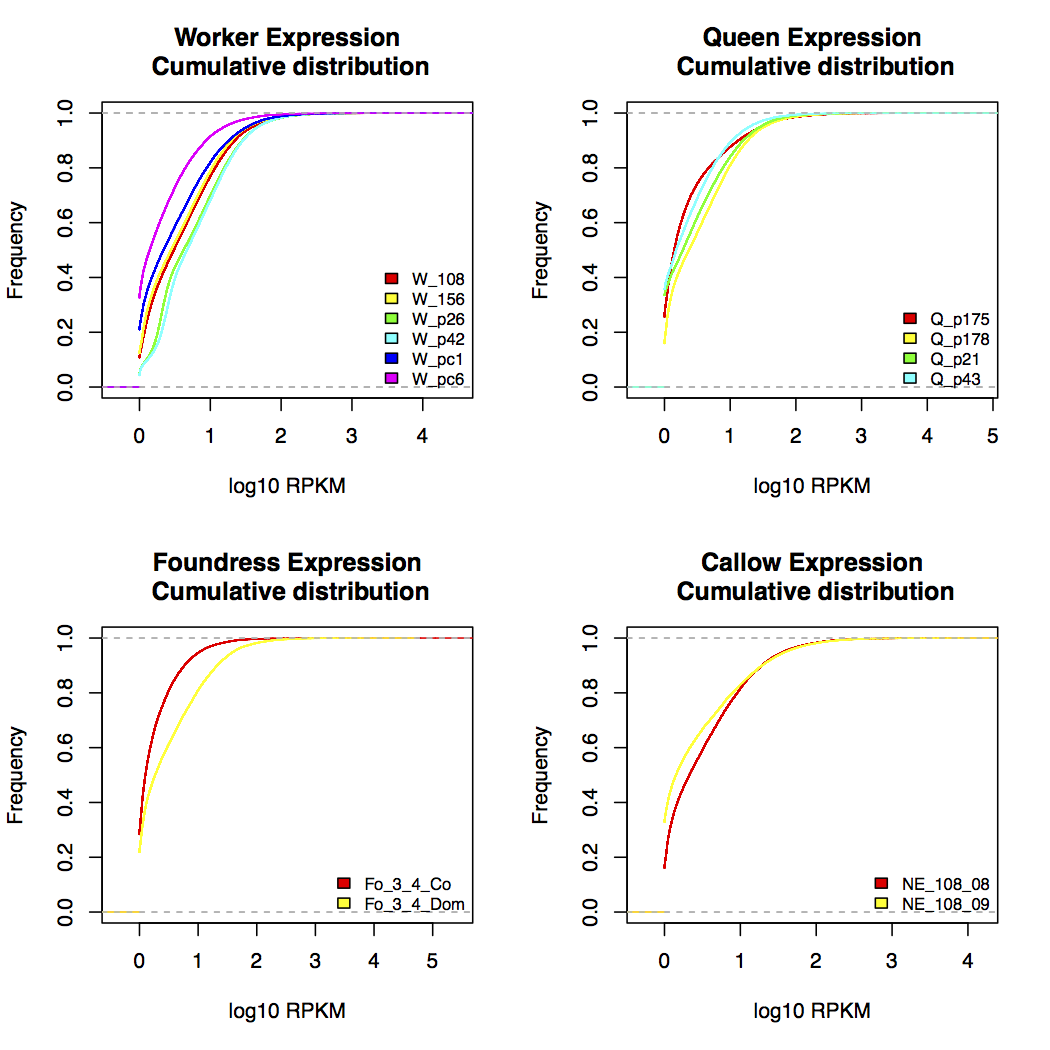

Supplement: Additional file 8 — Cumulative distribution of gene expression for the four phenotypes. [file gb-2013-14-2-r20-S8.TIFF]
